# Effects of Organizational Climate and Servant Leadership on Work Engagement Among Japanese Nurses: The Mediating Role of Psychological Safety

**DOI:** 10.1155/jonm/5755950

**Published:** 2026-09-01

**Authors:** Toshihiro Hontake, Masaki Nishi, Shingo Kuzushima, Naotoshi Kamizawa

**Affiliations:** ^1^ Department of Nursing, Faculty of Health Sciences, Kumamoto Health Science University, Kumamoto, Japan, kumamoto-hsu.ac.jp; ^2^ Department of Nursing, Faculty of Medical Technology, Teikyo University, Itabashi, Japan, teikyo-u.ac.jp; ^3^ Department of Nursing, Faculty of Nursing, Miyazaki Prefectural Nursing University, Miyazaki, Japan, mpu.ac.jp; ^4^ Division of Nursing Sciences, Graduate School of Human Health Sciences, Tokyo Metropolitan University, Arakawa, Tokyo, Japan, tmu.ac.jp

## Abstract

**Background:**

Work engagement is a pivotal concept associated with nurses’ occupational well‐being, performance, and retention. According to the job demands‐resources (JD‐R) model, work engagement is enhanced by job resources in the workplace. In nursing settings, hierarchical structures and interpersonal sensitivities may inhibit open expression, suggesting that psychological safety plays an important role in workplace interactions and work engagement. However, the mechanisms through which organizational climate and leadership influence work engagement via psychological safety have not been sufficiently examined.

**Aim:**

This study examined the mediating effect of psychological safety on the relationships between organizational climate, servant leadership, and work engagement among Japanese hospital nurses.

**Method:**

A questionnaire survey was conducted among nurses working in hospital wards in Japan. The study analyzed data on 306 participants, using the Japanese Utrecht Work Engagement Scale‐9, Japanese Psychological Safety Scale, Ward Organizational Climate Scale, and Servant Leadership Scale, Japanese version. Structural equation modeling was performed to examine the mediating effect of psychological safety.

**Results:**

Organizational climate had a significant positive association with psychological safety, which, in turn, was significantly associated with work engagement. Organizational climate showed a significant indirect association with work engagement through psychological safety. Servant leadership, however, did not demonstrate significant effects on either psychological safety or work engagement.

**Conclusions:**

These findings imply that organizational climate is associated with nurses’ work engagement through psychological safety. Within the tested model, organizational climate showed a stronger association with psychological safety than servant leadership. These findings also extend the application of the JD‐R model by suggesting that psychological safety may function as an interpersonal mechanism linking organizational climate to nurses’ work engagement.

**Implications for Nursing Management:**

The findings highlight the importance of prioritizing the development of a workplace environment that allows nurses to speak openly and seek consultation with confidence.

## 1. Introduction

Since the 2000s, work engagement has attracted increasing attention as a concept that enhances organizational performance, employees’ job performance, and organizational commitment [1–4]. Work engagement is conceptualized as a positive, fulfilling, work‐related state of mind characterized by vigor, dedication, and absorption [5]. According to the job demands‐resources (JD‐R) model, work engagement is shaped by the balance between job resources, such as support from colleagues and supervisors, and job demands, such as workload and emotional labor. When sufficient job resources are available, employees are more likely to experience intrinsic motivation, resulting in higher levels of work engagement [6].

Within the JD‐R framework, organizational and interpersonal job resources have received increasing attention because they determine how employees interact with others and engage in their work. These resources not only provide instrumental support but also create social environments that encourage communication, collaboration, and active participation in work‐related activities. Previous research has demonstrated that high‐quality interpersonal relationships in the workplace are essential for enhancing work engagement [7]. Therefore, identifying the interpersonal processes that mediate the relationship between job resources and work engagement has become an important issue in organizational research.

One important interpersonal process is psychological safety, defined as a shared belief among team members that the team is safe for interpersonal risk‐taking and that individuals can express their opinions, questions, and concerns without fear of negative evaluation [8]. Psychological safety is widely recognized as a key mechanism linking organizational factors, such as leadership and organizational climate, to employees’ attitudes and behaviors [9]. Within the JD‐R framework, psychological safety may function as a proximal interpersonal mechanism that translates organizational and interpersonal job resources into employees’ motivation and work engagement. Thus, organizational practices and managerial behaviors enhance work engagement, not directly but indirectly, by fostering psychologically safe work environments.

This perspective may be particularly relevant in Japanese nursing settings. Previous studies have reported that Japanese nurses tend to hesitate expressing their opinions because of concerns about potential interpersonal disadvantages associated with speaking up due to hierarchical relationships based on seniority and professional roles [10]. Such hierarchical workplace characteristics suggest that interpersonal risks are relatively high in Japanese nursing contexts and that psychological safety may therefore play a particularly important role in promoting communication, collaboration, and work engagement. Therefore, examining whether psychological safety mediates the relationship between job resources and work engagement is important for evaluating the applicability of the JD‐R model within Japanese nursing contexts.

Organizational climate can be understood as employees’ shared interpretations of workplace policies, practices, and procedures, along with behaviors that are encouraged, supported, and expected within the organization [11]. As a collective workplace resource, organizational climate reflects employees’ shared perceptions of what is valued within the organization and provides a social context that shapes interpersonal interactions and daily work experiences. Organizational climate has been shown to influence employees’ motivation and engagement [12, 13], and supportive and collaborative organizational climates have consistently been associated with higher levels of work engagement among nurses [14]. Furthermore, studies conducted in Japan have demonstrated that organizational justice, another organization‐level resource, is positively associated with work engagement [15]. These findings suggest that organization‐level resources contribute to employees’ motivation by establishing workplace environments characterized by trust, fairness, and mutual support.

Whether employees perceive interpersonal risk when expressing opinions or concerns likely depends on the organizational climate, reflecting shared workplace norms and expectations. Theoretically, organizational climate fosters psychological safety by creating environments in which speaking up and collaboration are accepted and supported.

In addition to organizational climate, leadership represents another important job resource within the JD‐R framework. Unlike organizational climate, which reflects collective workplace perceptions, leadership operates primarily through interpersonal interactions between supervisors and employees and therefore represents an individual‐level resource [16]. Previous research has shown that engaging leadership enhances intrinsic motivation, proactive energy, and work engagement by satisfying employees’ basic psychological needs [17]. Furthermore, leadership that emphasizes both task achievement and group maintenance among nurse managers has also been associated with higher work engagement [18].

The present study focuses on servant leadership because of its strong theoretical relevance to psychological safety, a feature that distinguishes it among different leadership approaches. Servant leadership emphasizes leaders’ commitment to supporting followers’ growth and well‐being [19]. In Japanese organizational settings, servant leadership is characterized by fostering others’ growth, humility, sincerity, trust, empathy, foresight, and commitment to community service [20]. This leadership behavior facilitates psychological safety by encouraging employees to express ideas, ask questions, and communicate concerns without fear of negative interpersonal consequences. In addition, servant leadership has been associated with employee resilience, perceived organizational support, and work engagement [21].

Although previous studies have demonstrated that organizational climate and leadership are each positively associated with work engagement, these factors have generally been examined independently. Consequently, little is known about their relative contributions to psychological safety and work engagement when considered simultaneously within the same theoretical framework. This represents an important theoretical gap because organizational climate and servant leadership constitute different types of job resources that operate at different levels of analysis. Organizational climate reflects shared organizational perceptions, whereas servant leadership reflects supervisors’ behavioral patterns toward individual employees. Determining whether these organization‐ and individual‐level resources contribute differently to psychological safety and work engagement is important for advancing the application of the JD‐R model.

At the same time, although organizational climate and servant leadership are conceptually distinct, they are likely to be closely related in practice because leadership behaviors contribute to shaping employees’ shared perceptions of the work environment. Accordingly, examining both constructs simultaneously may help clarify their respective associations with psychological safety and work engagement.

This theoretical understanding also has practical significance. Interventions to improve nurses’ work engagement often require substantial organizational investment, including leadership development, organizational reform, and workplace support systems. Therefore, understanding the relative contributions of different job resources may assist nursing managers in selecting evidence‐based strategies that efficiently enhance work engagement.

The need for such evidence is particularly important in contemporary healthcare settings, where workforce shortages, increasing job demands, and high turnover continue to challenge the sustainability of nursing organizations. Because nursing practice depends heavily on teamwork and interpersonal collaboration, both organizational environments and leadership behaviors may influence employees’ willingness to communicate openly and engage actively in their work.

Accordingly, this study proposes that organizational climate and servant leadership represent distinct job resources that contribute to work engagement through psychological safety. Following the JD‐R framework, psychological safety is conceptualized as an interpersonal mechanism linking these job resources to work engagement.

Examining these two types of job resources simultaneously may also advance understanding of the psychological safety mechanism by clarifying whether organizational‐ and individual‐level resources demonstrate independent, distinct, or overlapping associations with psychological safety and subsequent work engagement after accounting for one another.

Therefore, this study aimed to compare the relative contributions of an organizational‐level job resource (organizational climate) and an individual‐level job resource (servant leadership) to nurses’ work engagement through psychological safety within a single JD‐R framework. The study’s purpose was to compare their associations within the proposed model rather than to determine whether one job resource was inherently stronger than the other.

Building on this theoretical background, we formulated the following hypotheses:

 H1: Organizational climate is positively associated with psychological safety.
 H2: Servant leadership is positively associated with psychological safety.
 H3: Psychological safety is positively associated with work engagement.
 H4: Psychological safety mediates the relationships among organizational climate, servant leadership, and work engagement.

This study contributes to the literature in three ways. First, it simultaneously examines organizational climate and servant leadership within a unified JD‐R framework, enabling comparison of organization‐ and individual‐level job resources. Second, it clarifies the mediating role of psychological safety as an interpersonal mechanism linking job resources to work engagement. Third, it provides empirical evidence from Japanese nursing settings, thereby extending the application of the JD‐R model to a cultural context characterized by hierarchical workplace relationships and strong interpersonal norms.

## 2. Materials and Methods

### 2.1. Participants, Data Collection Procedure, and Study Period

Two public medical institutions and nine private medical institutions agreed to participate in the study, based on convenience sampling, executed through the principal investigator’s professional network. We first contacted the directors of nursing at each institution and explained the study’s purpose and procedures. After obtaining institutional agreement, they identified eligible participants according to predefined inclusion and exclusion criteria and distributed invitation letters containing a QR code linked to the web‐based questionnaire.

The study participants were registered staff nurses, assistant nurse managers, and nurse managers working in hospital wards across the 11 institutions—both general and psychiatric hospitals; nurses working in various wards (e.g., internal medicine, surgery, and psychiatry) were eligible.

The inclusion criteria were as follows: holding a registered nurse license; working in a hospital ward; having at least 1 year of nursing experience; and being employed full‐time. Participants who did not meet these criteria were excluded. In addition, directors of nursing, deputy directors of nursing, and nurse managers without responsibility for hospital wards were excluded because the purpose of this study was to investigate psychological safety and work engagement among ward‐based nurses and ward nurse managers. Nurses with less than 1 year of experience were also excluded because their limited clinical experience and familiarity with the ward environment might inhibit them from freely expressing their opinions within the workplace.

To minimize disproportionate representation from individual institutions, approximately 80–100 eligible nurses were recruited from each institution. All eligible nurses received an invitation letter, and participation was voluntary. Participants accessed an anonymous web‐based questionnaire by scanning a QR code and completed the survey at a time of their own choosing. Submission of the completed questionnaire was regarded as providing informed consent to participate in the study.

This study was conducted with the approval of the Ethics Review Committee of Fukuoka Jo Gakuin Nursing University (Approval No. 23‐10). The survey was administered between June and November 2024. All responses were collected anonymously, and appropriate measures were implemented to protect participants’ confidentiality.

### 2.2. Measures

#### 2.2.1. Demographic Characteristics

Participants were asked about sex, age group, years of nursing experience, total years of service at their current institution, and years of service in their current ward.

#### 2.2.2. Work Engagement

Work engagement was measured using the Japanese version of the Utrecht Work Engagement Scale‐9 (JUWES‐9), translated by Shimazu et al. [22] from the original Utrecht Work Engagement Scale (UWES‐9) developed by Schaufeli et al. [5]. The UWES consists of three subscales—vigor, dedication, and absorption—and includes nine items. Responses are rated on a seven‐point Likert scale ranging from 0 (“never”) to 6 (“always”). Regarding internal consistency, the Cronbach’s alpha coefficients for the three subscales of the original UWES‐9 developed by Schaufeli et al. ranged from 0.80 to 0.86 [5]. The translated JUWES‐9 also demonstrated high internal consistency, with a Cronbach’s alpha coefficient of 0.92 [22]. Permission to use this scale was obtained from the developer.

#### 2.2.3. Psychological Safety

Psychological safety was measured using the Japanese version of the Psychological Safety Scale (JPSS), translated by Ochiai and Otsuka [23] from the Psychological Safety Scale developed by Liang et al. [24]. This scale has a single‐factor structure and consists of five items. Responses are rated on a five‐point Likert scale ranging from 1 (“not at all applicable”) to 5 (“very applicable”). The Cronbach’s alpha coefficient has been reported to range from 0.88 to 0.91 [23]. Permission to use this scale was obtained from the developer.

#### 2.2.4. Organizational Climate

Individual nurses’ perceptions of organizational climate was measured using the Ward Organizational Climate Scale (WOCS) developed by Tsukamoto et al. [25]. This scale consists of four subscales—sense of control, staff morale, intimacy, and learning atmosphere—and includes 14 items. Responses are rated on a five‐point Likert scale ranging from 1 (“not at all true”) to 5 (“always true”) [25].

The Cronbach’s alpha coefficients for the subscales ranged from 0.73 to 0.87. Permission to use this scale was obtained from the developer.

#### 2.2.5. Leadership

Servant leadership was measured using the 28‐item Japanese version of the Servant Leadership Scale (SLJ‐28) translated by Kiuchi and Osaki [26] from the SLJ developed by Liden et al. [16]. The SLJ‐28 includes the following seven subscales: conceptual skills, empowering, helping subordinates grow and succeed, putting subordinates first, behaving ethically, emotional healing, and creating value for the community. Responses are rated on a seven‐point Likert scale ranging from 1 (“not at all applicable”) to 7 (“very applicable”). Regarding internal consistency, the original version reported Cronbach’s alpha coefficients ranging from 0.86 to 0.91 across subscales. For the SLJ‐28, the seven‐factor model also demonstrated adequate fit (comparative fit index [CFI] = 0.951, root mean square error of approximation [RMSEA] = 0.045, SRMR = 0.052), and Cronbach’s alpha coefficients were generally 0.80 or higher. Permission to use this scale was obtained from the developer.

### 2.3. Statistical Analysis

#### 2.3.1. Analytical Sample

An anonymous self‐administered questionnaire survey was conducted with 850 nurses working in 11 medical institutions who met the inclusion criteria. Responses were obtained from 309 participants (response rate: 36.0%). Of these, three participants were excluded because of inappropriate responses to questions on basic attributes, resulting in a final analytical sample of 306 participants (valid response rate: 99.0%). No data were missing among the 306 valid responses; therefore, complete‐case analysis was performed. Because convenience sampling was used, and the response rate was relatively low (36.0%), the sample may not fully represent the broader population of nurses, which should be considered when interpreting the generalizability of this study’s findings.

#### 2.3.2. Reliability of the Scales

Cronbach’s alpha coefficients were calculated to confirm the internal consistency of the scales used in this study. In addition, composite reliability (CR) and average variance extracted (AVE) were calculated to evaluate the reliability and convergent validity of the latent constructs included in the structural equation model.

#### 2.3.3. Examination of Background Factors

The background factors examined included age, years of nursing experience, years of service at the current institution, and years of service in the current ward. Correlation analyses were conducted to examine the relationships between these background factors and the JUWES‐9, JPSS, SLJ‐28, and WOCS scores. Correlation coefficients were interpreted as follows: < 0.10, negligible; 0.10–0.29, weak; 0.30–0.49, moderate; and ≥ 0.50, strong.

#### 2.3.4. Assessment of Normality

Data were screened prior to the main analyses, using boxplots, normal Q‐Q plots, and the Shapiro–Wilk test, to examine outliers and normality. No substantial outliers or serious violations of normality were identified. The results indicated significant nonnormality for all scales. However, the Shapiro–Wilk test may indicate statistical significance even with slight deviations from normality in large samples [27]. Therefore, analytical procedures robust to nonnormality were adopted in subsequent analyses.

#### 2.3.5. Structural Equation Modeling

In this study, work engagement, psychological safety, servant leadership, and organizational climate were incorporated into a SEM framework as latent variables representing multidimensional psychological and organizational phenomena. SEM was employed because it enables the simultaneous estimation of complex relationships among latent constructs while accounting for measurement error.

Modeling these constructs as latent variables rather than using total scores allows relationships among constructs to be estimated while accounting for measurement error.

The following subscales were used as observed indicators for each latent variable: *work engagement* was specified using the three subscales of vigor, dedication, and absorption; *organizational climate* was specified using the four subscales of sense of control, staff morale, intimacy, and learning atmosphere; and *servant leadership* was specified using the seven subscales of the SLJ‐28. Because psychological safety was measured using a single‐factor scale, individual item scores were used as indicators of the latent variable.

The use of latent variables in SEM minimizes the influence of measurement error and enables more precise estimation of theoretical relationships among constructs [28]. In addition, using subscales as indicators allows relationships among constructs to be tested while maintaining the stability of the measurement model. Constructs commonly examined in nursing management research (e.g., work engagement, psychological safety, leadership, and organizational climate) are abstract and cannot be adequately captured by a single indicator; therefore, they are widely modeled as latent variables [29]. In this study, organizational climate was operationalized as individual nurses’ perceptions of organizational climate because all variables were collected through individual self‐report questionnaires.

Prior to testing the structural model, confirmatory factor analysis (CFA) was conducted to evaluate the measurement model. CR and AVE were calculated to assess the reliability and convergent validity of the latent constructs. Multicollinearity among predictor variables was examined using tolerance and variance inflation factor values.

Maximum likelihood estimation was used for model estimation. Model fit was evaluated using the chi‐square test (*χ* ^2^), CFI, Tucker–Lewis index (TLI), and RMSEA. The criteria for acceptable model fit were CFI and TLI values of 0.90 or higher and RMSEA values of 0.08 or lower, whereas values of 0.95 or higher and 0.05 or lower, respectively, were considered indicative of good fit [30].

Based on the recommendations of Westland [31]—that sample size in SEM should be evaluated according to model complexity rather than by applying a universal cutoff—the present sample size (*N* = 306) was considered adequate for the complexity of the proposed model [31].

#### 2.3.6. Bootstrap Estimation

The bootstrap method was used to ensure the robustness of estimates under nonnormal distribution conditions and to test the significance of mediating effects. Bootstrap resampling was performed 5000 times, using bias‐corrected 95% confidence intervals, and indirect effects were considered significant when the 95% confidence interval did not include zero. The bootstrap method does not depend on assumptions about population distribution and has been shown to be particularly effective for testing indirect effects [32].

All descriptive and inferential statistical analyses were conducted using IBM SPSS Statistics version 29 (IBM Corp., Armonk, NY, USA). SEM and bootstrap mediation analyses were conducted using IBM SPSS Amos version 31 (IBM Corp., Armonk, NY, USA). Statistical significance was assessed using a two‐sided significance level of *α* = 0.05.

## 3. Results

### 3.1. Participants’ Characteristics and Descriptive Statistics of the Study Variables

Table 1 presents the demographic characteristics of the participants and descriptive statistics for each scale. The study sample comprised 306 nurses; of these, 273 were women (89.2%), 32 were men (10.5%), and 1 participant reported other or did not respond (0.3%). The mean age of the participants was 39.05 years (SD = 11.73), and the mean number of years of nursing experience was 16.04 years (SD = 10.95). The mean length of service at the current institution was 10.62 years (SD = 8.89), and the mean length of service in the current ward or department was 4.58 years (SD = 3.57).

**TABLE 1:** Participant characteristics and descriptive statistics (*N* = 306).

Variable	Min	Max	Mean	SD
Age (years)	21	64	39.05	11.73
Nursing experience (years)	1	44	16.04	10.95
Tenure in current organization (years)	1	44	10.62	8.89
Tenure in current ward/unit (years)	1	20	4.58	3.57

*Sex/Gender, n (%)*				
Female	273	89.2 (%)		
Male	32	10.5 (%)		
Other/prefer not to say	1	0.3 (%)		

*Scale*				
JUWES‐9	0	54	21.10	10.20
JPSS	6	23	15.27	3.54
SLJ‐28	28	194	121.53	28.14
WOCS	14	70	47.08	8.26

*Note:* JUWES‐9 = Japanese version of the Utrecht Work Engagement Scale‐9; SLJ‐28 = the 28‐item Japanese version of the Servant Leadership Scale.

Abbreviations: JPSS = Japanese Psychological Safety Scale, WOCS = Ward Organizational Climate Scale.

The mean scores (SD) for each scale were as follows: JUWES‐9, 21.10 (SD = 10.20); JPSS, 15.27 (SD = 3.54); SLJ‐28, 121.53 (SD = 28.14); and WOCS, 47.08 (SD = 8.26). Cronbach’s alpha coefficients in this study were 0.94 for JUWES‐9, 0.81 for JPSS, 0.97 for SLJ‐28, and 0.90 for WOCS, indicating good internal consistency for all scales. In addition, CR values ranged from 0.804 to 0.952, and AVE ranged from 0.493 to 0.781, indicating acceptable reliability.

### 3.2. Relationships Between Background Factors and Study Variables

Table 2 presents the relationships between background factors and study variables. JUWES‐9 showed significant moderate positive correlations with JPSS (*r* = 0.34, *p* < 0.001), SLJ‐28 (*r* = 0.33, *p* < 0.001), and WOCS (*r* = 0.32, *p* < 0.001). In contrast, weak positive correlations were observed with age (*r* = 0.14, *p* = 0.017) and years of nursing experience (*r* = 0.13, *p* = 0.024), whereas no significant associations were found with years of service at the current institution or years of service in the current ward.

**TABLE 2:** Intercorrelations among the study variables.

Variable	1	2	3	4
1. JUWES‐9	1			
2. JPSS	0.34	1		
3. SLJ‐28	0.33	0.49	1	
4. WOCS	0.32	0.59	0.61	1

*Note:* All correlations are significant at *p* < 0.001. Abbreviations are explained in Table 1.

JPSS showed moderate to strong positive correlations with SLJ‐28 (*r* = 0.49, *p* < 0.001) and WOCS (*r* = 0.59, *p* < 0.001). SLJ‐28 was also significantly correlated with WOCS (*r* = 0.61, *p* < 0.001). Table 3 presents the relationships between each variable and age and years of service; however, no significant associations were found between JPSS and any of the variables.

**TABLE 3:** Associations between participants’ background characteristics and the study variables (*N* = 306).

Variable	Age	Nursing experience	Tenure in current organization	Tenure in current ward/unit
JUWES‐9	0.14	0.13	0.04	0.02
JPSS	−0.03	0.00	0.01	−0.02
SLJ‐28	−0.17	−0.14	−0.13	−0.09
WOCS	−0.23	−0.20	−0.18	−0.13

*Note:* Pearson’s correlations. Abbreviations are explained in Table 1.

Overall, these associations supported the appropriateness of testing the hypothesized structural model.

### 3.3. Preliminary Analyses

Prior to SEM, preliminary analyses were conducted to examine missing data, common method bias, data screening, and differences among ward types. No data were missing among the 306 valid responses. Data screening using boxplots, normal Q‐Q plots, and the Shapiro–Wilk test revealed no substantial outliers or serious violations of normality. Harman’s single‐factor test indicated that the first unrotated factor accounted for 34.8% of the total variance, suggesting that common method bias most likely did not substantially affect the findings.

One‐way analysis of variance showed a significant difference in work engagement among ward types (*F*(6,299) = 2.764, *p* = 0.012, *η* ^2^ = 0.053). Post hoc Tukey tests indicated significantly higher work engagement among nurses working in obstetrics/pediatrics wards than among those working in internal medicine wards (*p* = 0.030) and convalescent/rehabilitation wards (*p* = 0.017). No significant differences were observed among ward types for psychological safety (*F* = 0.603, *p* = 0.728), servant leadership (*F* = 0.778, *p* = 0.588), or organizational climate (*F* = 0.360, *p* = 0.904).

### 3.4. SEM of Servant Leadership, Organizational Climate, Psychological Safety, and Work Engagement

**FIGURE 1:**
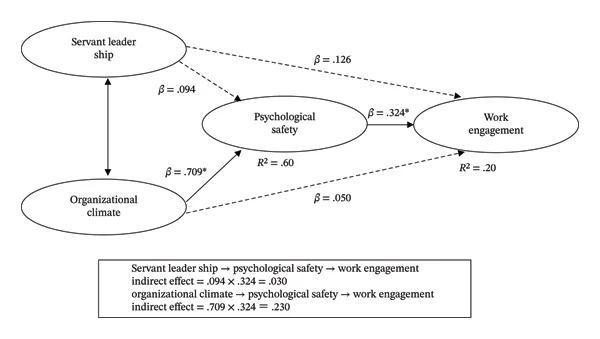
Structural model of the relationships among servant leadership, organizational climate, psychological safety, and work engagement. Solid lines indicate statistically significant paths (*p* < 0 05), and dashed lines indicate nonsignificant paths. Values represent standardized path coefficients. *R* ^2^values are shown for endogenous variables.

Table 4 and Figure 1 present an SEM of servant leadership, organizational climate, psychological safety, and work engagement. The following subsections describe model fit, direct effects, and indirect effects of the SEM.

**TABLE 4:** Results of structural equation modeling.

Direct effects path	Standardized β	95% CI lower	95% CI upper	*p* value
OC ⟶ PS	0.71	0.52	0.89	< 0.001
SL ⟶ PS	0.09	−0.10	0.28	0.324
PS ⟶ WE	0.32	0.08	0.58	0.009
OC ⟶ WE	0.05	−0.27	0.34	0.759
SL ⟶ WE	0.13	−0.08	0.34	0.223

**Indirect effects path (boot strap, bias-corrected 95% CI)**	**Standardized β**	**Unstandardized estimate**	**95% CI lower**	**95% CI upper**

OC ⟶ PS ⟶ WE	0.23	0.362	0.058	1.030
SL ⟶ PS ⟶ WE	0.03	0.03	−0.03	0.108
*R* ^2^ Psychological safety	0.60			
*R* ^2^ Work engagement	0.20			

*Model fit indices*				
*χ* ^2^(146)	385.70			
*p* value	< 0.001			
CFI	0.942			
TLI	0.93			
RMSEA	0.07			
RMSEA 90% CI lower	0.07			
RMSEA 90% CI upper	0.08			

*Note:* RMSEA = root mean square error of approximation.

Abbreviations: CFI = comparative fit index, OC = organizational climate, PS = psychological safety, SL = servant leadership, TLI = Tucker–Lewis index, WE = work engagement.

#### 3.4.1. Measurement Model

Prior to testing the structural model, CFA was conducted to evaluate the adequacy of the measurement model. The measurement model demonstrated acceptable fit to the data (*χ* ^2^(146) = 385.701, *p* < 0.001, CFI = 0.942, TLI = 0.932, GFI = 0.882, AGFI = 0.846, and RMSEA = 0.073). Standardized factor loadings ranged from 0.631 to 0.752 for organizational climate, 0.774 to 0.917 for servant leadership, 0.507 to 0.881 for psychological safety, and 0.845 to 0.911 for work engagement. CR ranged from 0.804 to 0.952, and AVE ranged from 0.493 to 0.781, supporting the adequacy of the measurement model.

#### 3.4.2. Model Fit of the Structural Model

The structural model with psychological safety as a mediator demonstrated acceptable model fit: *χ* ^2^ (146) = 385.701, *p* < 0.001, CFI = 0.942, TLI = 0.932, and RMSEA = 0.073 (90% confidence interval: 0.065–0.082). The coefficients of determination indicated that the *R* ^2^ value for psychological safety was 0.603, whereas the *R* ^2^ value for work engagement was 0.204. These coefficients indicate that the model explained 60.3% of the variance in psychological safety and 20.4% in work engagement.

#### 3.4.3. Direct Effects

The standardized estimates indicated that organizational climate had a significant positive effect on psychological safety (*β* = 0.709, *p* < 0.001). In contrast, servant leadership did not have a significant effect on psychological safety (*β* = 0.094, *p* = 0.324). Psychological safety had a significant positive effect on work engagement (*β* = 0.324, *p* = 0.009). The direct effect of organizational climate on work engagement was not significant (*β* = 0.050, *p* = 0.759), and servant leadership did not have a significant effect on work engagement (*β* = 0.126, *p* = 0.223). Thus, only the organizational climate‐psychological safety and psychological safety‐work engagement paths were statistically significant.

#### 3.4.4. Indirect Effects

The indirect effect of organizational climate on work engagement through psychological safety was significant. The standardized indirect effect was *β* = 0.230, and the bias‐corrected 95% bootstrap confidence interval for the unstandardized indirect effect ranged from 0.058 to 1.030, not including zero. These findings indicate that psychological safety significantly mediated the relationship between organizational climate and work engagement.

In contrast, the indirect effect of servant leadership on work engagement through psychological safety was not significant. The standardized indirect effect was *β* = 0.030, and the 95% bootstrap confidence interval for the unstandardized indirect effect included zero. Therefore, no significant mediating effect of psychological safety was found between servant leadership and work engagement.

## 4. Discussion

This study examined the effects of organizational climate and servant leadership on nurses’ work engagement using a structural model in which psychological safety served as a mediating variable.

Although this study was conducted among nurses working in Japanese healthcare institutions, psychological safety and organizational climate are internationally recognized as key constructs shaping nursing work environments and patient safety culture [10, 14]. In recent years, global nursing shortages and high turnover have become increasingly critical issues, making the identification of organizational factors that enhance work engagement an international priority. Therefore, although the present findings are grounded in Japanese nursing contexts, the constructs of psychological safety, organizational climate, and work engagement are widely applicable across countries. Accordingly, the findings may offer theoretical implications for nursing management practices beyond Japan.

The results supported H1, H3, and H4, indicating that organizational climate was significantly associated with psychological safety and that work engagement was related to organizational climate through psychological safety. In contrast, H2 was not supported, as servant leadership did not show significant associations with either psychological safety or work engagement. These results suggest that individual nurses’ perceptions of organizational climate are associated with their work engagement through psychological safety within the tested model. However, because this study employed a cross‐sectional design, causal relationships cannot be established, and the observed associations should be interpreted as model‐based relationships rather than directional effects.

In addition, although the model explained a substantial proportion of the variance in psychological safety (*R* ^2^ = 0.603), it explained a more modest proportion of the variance in work engagement (*R* ^2^ = 0.204). This finding suggests that work engagement is also influenced by additional individual‐, occupational‐, and organizational‐level factors that were not included in the present model, and which should be examined in future studies.

The absence of significant associations between servant leadership and psychological safety or work engagement may be explained by the distinction between individual‐ and collective‐level constructs. Leadership is typically perceived as the behavior of individual supervisors, whereas psychological safety represents a shared belief formed through team interactions and collective norms. Thus, in nursing workplaces, the formation of psychological safety may be more strongly related to organizational factors (e.g., shared climate and peer interactions) than to the behavior of individual leaders alone.

Furthermore, nursing workplaces are not structured solely around dyadic supervisor–subordinate relationships; rather, they function as collective environments in which work is conducted through interactions with multiple supervisors, colleagues, and interprofessional team members. In such contexts, organizational climate—as a shared perception formed through everyday interactions—may play a more fundamental role in shaping psychological safety than the leadership of a single manager.

These findings do not negate the importance of leadership but suggest the possibility that leadership effects may emerge indirectly through the formation of organizational climate; however, this hypothesized pathway was not directly examined in this study. Future studies should explicitly examine this hypothesized chained pathway using longitudinal or intervention designs to determine whether organizational climate mediates the relationship between servant leadership and psychological safety or work engagement. When organizational climate and servant leadership are included simultaneously within the same model, organizational climate—as a broader and more comprehensive construct—may account for a larger proportion of variance in psychological safety, thereby reducing the unique contribution of servant leadership to nonsignificant levels.

The present findings suggest that organization‐level interventions targeting shared workplace climate are more important for fostering psychological safety and enhancing nurses’ work engagement than interventions focusing solely on individual leadership. However, this interpretation should be made cautiously, as SEM results reflect relationships within a specified model and do not necessarily indicate the relative importance of constructs across different contexts.

While previous studies have primarily examined leadership and work engagement within a supervisor–subordinate framework, this study extends the literature by demonstrating that organization‐level interactions are associated with work engagement through the mediating mechanism of psychological safety. Moreover, within the JD‐R model framework, the finding that organizational climate is associated with work engagement through psychological safety suggests that both organizational climate and psychological safety function as important job resources for nurses.

### 4.1. Effects of Organizational Climate on Work Engagement

A key finding of this study is that psychological safety mediates the relationship between organizational climate and work engagement. In particular, the effect of organizational climate on perceived psychological safety was relatively strong (*β* = 0.709). This may reflect the close conceptual relationship between psychological safety and shared perceptions of workplace interactions and communication patterns. Perceived organizational climate—such as perceived workplace values and a climate of mutual support—may play a central role in shaping nurses’ psychological safety by enabling them to express their opinions without fear.

However, the magnitude of this coefficient warrants careful interpretation. Because both organizational climate and psychological safety include elements related to interpersonal relationships and workplace interactions, their conceptual proximity may have contributed to the relatively strong association observed in this study. Thus, the observed relationship may partially reflect conceptual overlap rather than a purely distinct effect between constructs. Nevertheless, these constructs remain theoretically distinct. Organizational climate represents a shared, group‐level perception of workplace values and behavioral norms, whereas psychological safety reflects an individual‐level perception of being able to take interpersonal risks within a team [9, 11]. In addition, although organizational climate is inherently a group‐level construct, it was measured at the individual level in this study, which may have influenced the strength of the observed associations. The measurement model demonstrated acceptable construct reliability and measurement validity. Accordingly, the structural model should be interpreted in light of its acceptable, rather than excellent, overall fit.

Therefore, the present findings do not undermine the discriminant validity of these constructs. Rather, they suggest that psychological safety is conceptualized as a more proximal construct that develops within the broader contextual framework of organizational climate.

Moreover, the correlation between organizational climate and servant leadership was moderate (*r* = 0.61) and did not reach the threshold typically associated with multicollinearity concerns (*r* ≥ 0.80). This indicates that the observed relationship is unlikely to be a statistical artifact and instead reflects a meaningful substantive association. Organization‐level contextual factors appear to be closely related to the development of psychological safety.

Organizational climate is formed and sustained through daily workplace interactions, reflecting shared values, behavioral norms, and communication patterns among staff [11]. Behaviors such as speaking openly, reporting incidents, and expressing concerns inherently involve interpersonal risk, as they may challenge professional relationships or hierarchical structures [33]. Prior research has shown that Japanese nursing workplaces are often characterized by hierarchical organizational structures and a cultural emphasis on harmony, which may discourage nurses from expressing opinions due to interpersonal considerations [10].

Although Japan is frequently described as having collectivistic and hierarchical cultural characteristics, the strong association observed between organizational climate and psychological safety suggests that structural and organizational interventions remain highly influential despite such cultural contexts. These findings imply that organization‐level strategies may be particularly important in hierarchical healthcare environments and highlight the need for further validation across different cultural and institutional settings.

In environments characterized by high interpersonal risk, nurses may hesitate to voice opinions, and shared perceptions of risk may contribute to stagnation in workplace interactions [33]. Reduced openness in communication may hinder the development of psychological safety. However, because this study employed a cross‐sectional design, causal interpretations should be made cautiously.

Previous studies have demonstrated that adequate staffing and organizational support positively influence communication within healthcare teams [34]. In Japan, nursing workplaces frequently experience chronic staff shortages and heavy workloads [35]. High workload levels have been associated with reduced teamwork and increased missed nursing care [36]. Under conditions requiring emergency responses or multitasking, nurses may have limited opportunities for communication and mutual support, further constraining workplace interaction. Such stagnation may weaken interpersonal trust within the organizational climate and consequently undermine psychological safety. These findings are consistent with the JD‐R model, which posits that excessive job demands, such as workload and time pressure, increase psychological strain and reduce work engagement. From this perspective, organizational climate and psychological safety can be understood as critical job resources that support nurses’ engagement.

The coefficient of determination for psychological safety was relatively high (*R* ^2^ = 0.603), indicating that organizational climate and servant leadership together explained a substantial proportion of its variance. This finding suggests that psychological safety among nurses is shaped not only by individual characteristics but largely by workplace environmental and organizational conditions.

Organizational climate is also influenced by managerial behaviors, institutional policies, and decision‐making processes [11], underscoring the important role of nurse managers in shaping ward‐level climate. Nurse managers are responsible for maintaining quality and safety in nursing practice, including staffing allocation, task coordination, educational support, and work environment management [37]. Although leadership contributes to fostering environments that enable open communication, reliance solely on individual managerial capabilities may be insufficient to explain the emergence of psychological safety. The nonsignificant effect of servant leadership on psychological safety suggests that leadership alone does not fully account for its development.

Nurse managers occupy a central coordinating role within hospital wards, managing interpersonal relationships among staff while collaborating with physicians and other healthcare professionals. This role entails substantial emotional labor and psychological burden [38, 39]. Managers frequently experience role conflict and sustained interpersonal stress while mediating between frontline nurses, physicians, and senior administration [38, 39]. Managerial stress has also been associated with workplace environmental factors and interpersonal conflict [40], indicating that relational coordination represents a major source of occupational strain.

Accordingly, nurse managers function as key agents in shaping organizational climate while simultaneously operating as middle managers facing multiple role demands. Leadership has consistently been linked to workplace environments and staff outcomes [41]; therefore, enabling nurse managers to exercise leadership under conditions of adequate organizational support is likely to facilitate the development of psychologically safe organizational climates. Evidence showing associations between nurse manager job satisfaction, turnover intentions, workload, and organizational support further suggests that excessive role burdens negatively influence team management and climate formation [37].

Creating organizational climates that encourage open communication among nurses requires not only effective individual leadership but also organizational systems that sustain workplace interaction and collaboration. This interpretation aligns with the present finding that organizational climate exerted a significant positive effect on psychological safety.

Collectively, these findings indicate that the development of psychologically safe workplaces depends on both leadership and structural organizational support. Establishing environments in which nurse managers are supported through institutional resources, professional development opportunities, and manageable role expectations may promote collaborative interactions and foster psychologically safe nursing workplaces.

### 4.2. Servant Leadership

Previous studies have supported the view that nurse managers’ leadership influences work engagement [42]. However, in this study, servant leadership did not show significant association with either psychological safety or work engagement.

The effect of servant leadership on psychological safety was not significant; therefore, H2 was not supported. One possible explanation for this inconsistency with previous research is that servant leadership depends on subordinates’ subjective perceptions, and its effects may be more susceptible to collective factors such as overall workplace interactions and shared norms [21]. From this perspective, the findings suggest that the servant leadership of a specific manager alone does not sufficiently explain psychological safety.

Servant leadership is characterized as a leadership behavior that fosters trust and collaboration through support, respect, and the promotion of subordinates’ growth. In this regard, servant leadership may function less as a direct determinant of psychological safety and more as a foundational factor contributing to the formation of organizational climate through the accumulation of everyday interactions. Consequently, it may be indirectly associated with psychological safety and work engagement through its influence on workplace climate.

Because organizational climate and servant leadership were simultaneously entered into the SEM model, organizational climate showed a stronger association with psychological safety within the tested model. However, because alternative model specifications (e.g., models including only servant leadership) were not examined, this finding should be interpreted as reflecting the relationships observed in the present model rather than definitive evidence that organizational climate attenuated the contribution of servant leadership.

A moderate correlation was observed between servant leadership and organizational climate (*r* = 0.61), suggesting a degree of conceptual overlap between the two constructs. However, this value did not reach the level generally considered indicative of problematic multicollinearity (*r* ≥ 0.80). Therefore, although the findings indicate relatedness between the constructs, they do not suggest statistical multicollinearity. Instead, the explanatory power of organizational climate may have been relatively greater because SEM estimates the independent contribution of each variable when both are included in the same model.

When leadership and organizational climate are examined within the same model, organizational climate—formed through the accumulation of individual leadership behaviors and daily interactions—may operate as a higher‐order contextual construct and therefore have a stronger association with psychological safety than individual leadership behavior. Accordingly, these findings suggest that individual leadership behavior alone does not sufficiently explain the formation of psychological safety or work engagement.

Furthermore, nursing workplaces are not composed solely of dyadic supervisor–subordinate relationships; rather, they are collective environments in which work is conducted through interactions with multiple supervisors, colleagues, and interprofessional staff [10]. Therefore, beyond perceptions of a specific manager’s leadership, organizational climate—as a shared workplace perception formed through routine interactions—may play a more fundamental role in shaping psychological safety.

Importantly, these findings do not negate the importance of leadership. Rather, they suggest that leadership effects manifest indirectly through the formation of organizational climate. Future research should examine the extent of conceptual overlap between organizational climate and servant leadership more rigorously and further evaluate measurement validity, discriminant validity, and collinearity within the measurement model. Longitudinal and multilevel designs are also needed to clarify causal directions and disentangle the roles of individual‐ and group‐level factors.

## 5. Implications for Nursing Management

Although these management implications are based on the associations observed in the present cross‐sectional study, they should be further examined through longitudinal and intervention studies to strengthen the evidence supporting these recommendations.

This study’s findings highlight the importance of an organizational climate that supports psychological safety in enhancing nurses’ work engagement. Accordingly, nursing managers should not rely solely on individual nurses’ efforts or isolated leadership training programs; rather, they should prioritize developing workplace environments in which nurses can speak openly and seek consultation with confidence.

Nurse managers play an important role in shaping such an organizational climate. However, achieving this goal should not depend solely on the personal capabilities of individual nurse managers.

## 6. Limitations

This study focused on nurses working in Japanese healthcare institutions; therefore, caution is required when generalizing the findings to other countries with different cultural and organizational contexts. In addition, given the study’s cross‐sectional design, causal relationships among the variables should be interpreted cautiously. Furthermore, participants were recruited using convenience sampling from 11 healthcare institutions, and institution‐specific sample distributions were not reported because the study was designed to examine individual‐ rather than institution‐level relationships. Consequently, potential institutional clustering was not explicitly examined. Future studies should report institution‐level sample distributions and incorporate multilevel analytical approaches to further evaluate clustering effects and strengthen the generalizability of the findings.

This study was based on individual‐level perceptual data, and organizational climate—originally conceptualized as a group‐level construct—was operationalized and analyzed as individual nurses’ perceptions of organizational climate, representing a methodological limitation. Therefore, the findings should be interpreted as reflecting perceived organizational climate rather than an aggregated organizational climate. Future studies should employ multilevel analyses or collect ward‐level data to capture group‐level effects more appropriately.

Because all variables were measured using self‐administered questionnaires at a single time point, the results may be subject to response bias and common method bias. Although Harman’s single‐factor test suggested that common method bias was unlikely to substantially influence the findings, this test alone cannot completely exclude common method variance. Future studies should employ more rigorous approaches, such as a common latent factor or marker‐variable technique, to further evaluate the potential influence of common method variance. Moreover, because convenience sampling was used, the representativeness of the sample may be limited. Despite these limitations, the present findings provide empirical support for the proposed theoretical framework.

Future researchers should validate the proposed model using longitudinal and multilevel designs across multiple institutions and cultural contexts. They should further examine causal relationships between organizational factors that enhance psychological safety and nurses’ work engagement using longitudinal and intervention designs and evaluate the effectiveness of nursing management interventions targeting organizational climate.

## 7. Conclusion

This study demonstrated that organizational climate was positively associated with nurses’ work engagement through psychological safety, whereas servant leadership did not show significant direct or indirect associations within the tested model. These findings suggest that psychological safety functions as an important interpersonal mechanism linking organization‐level job resources to work engagement, thereby extending the application of the JD‐R model in Japanese nursing settings. From a nursing management perspective, fostering psychologically safe work environments through supportive organizational climates may be more effective for enhancing nurses’ work engagement than focusing solely on individual leadership behaviors. Future longitudinal and multilevel studies are warranted to confirm these relationships and further clarify the organizational processes underlying psychological safety and work engagement.

## Author Contributions

Toshihiro Hontake contributed to the conception of the study, ethical approval procedures, data collection, analysis, and drafting of the manuscript. Naotoshi Kamizawa, Masaki Nishi, and Shingo Kuzushima contributed to the study conception, interpretation of the results, and drafting and revision of the manuscript.

## Funding

This work was supported by JSPS KAKENHI (grant number JP23K16410).

## Ethics Statement

This study was approved by the Ethics Review Committee of Fukuoka Jo Gakuin Nursing University (Approval No. 23‐10). Prior to requesting cooperation from the participating institutions, a written explanation of the study was provided, and institutional consent was obtained following the prescribed procedures at each site. For individual participants, a study information sheet including a QR code linking to the web‐based questionnaire was distributed via nurse managers at each institution. Informed consent was obtained using a consent confirmation item within the online questionnaire.

## Conflicts of Interest

The authors declare no conflicts of interest.

## Data Availability

The data supporting the findings of this study are available from the corresponding author upon reasonable request.
